# Lower amygdala fatty acid amide hydrolase in violent offenders with antisocial personality disorder: an [^11^C]CURB positron emission tomography study

**DOI:** 10.1038/s41398-020-01144-2

**Published:** 2021-01-18

**Authors:** Nathan J. Kolla, Isabelle Boileau, Karolina Karas, Jeremy J. Watts, Pablo Rusjan, Sylvain Houle, Romina Mizrahi

**Affiliations:** 1 Centre for Addiction and Mental Health (CAMH), Toronto, ON Canada; 2 Violence Prevention Neurobiological Research Unit, CAMH, Toronto, ON Canada; 3 Department of Psychiatry, University of Toronto, Toronto, ON Canada; 4 Department of Pharmacology and Toxicology, University of Toronto, Toronto, ON Canada; 5 Waypoint Centre for Mental Health Care, Penetanguishene, ON Canada; 6 McGill University, Montreal, QC Canada

**Keywords:** Diagnostic markers, Schizophrenia

## Abstract

Antisocial personality disorder (ASPD) imposes a high societal burden given the repetitive reactive aggression that affected individuals perpetrate. Since the brain endocannabinoid system (ECS) has been implicated in ASPD and aggressive behavior, we utilized [^11^C]CURB positron emission tomography to investigate fatty acid amide hydrolase (FAAH), an enzyme of the ECS that degrades anandamide, in 16 individuals with ASPD and 16 control participants. We hypothesized that FAAH density would be lower in the amygdala for several reasons. First, decreased FAAH expression is associated with increased cannabinoid receptor 1 stimulation, which may be responsible for amygdala hyper-reactivity in reactive aggression. Second, the amygdala is the seat of the neural circuit mediating reactive aggression. Third, other PET studies of externalizing populations show reduced brain FAAH density. Conversely, we hypothesized that FAAH expression would be greater in the orbitofrontal cortex. Consistent with our hypothesis, we found that amygdala FAAH density was lower in the amygdala of ASPD (*p* = 0.013). Cerebellar and striatal FAAH expression were inversely related with impulsivity (cerebellum: *r* = −0.60, *p* = 0.017; dorsal caudate: *r* = −0.58, *p* = 0.023; dorsal putamen: *r* = −0.55, *p* = 0.034), while cerebellar FAAH density was also negatively associated with assaultive aggression (*r* = −0.54, *p* = 0.035). ASPD presents high levels of disruptive behavior with few, if any, efficacious treatment options. Novel therapeutics that increase FAAH brain levels in a region-specific manner could hold promise for attenuating certain symptom clusters of ASPD, although our results require replication.

## Introduction

Antisocial personality disorder (ASPD) is a neurodevelopmental condition characterized by reckless, aggressive, and impulsive behavior^1^. Half of all individuals with ASPD possess a record of criminal offending, and 85% have acted violently toward others^2,3^. ASPD is also associated with the highest risk of victimizing children and strangers among all psychiatric disorders^4^. Many mental disorders are often comorbid with ASPD, such as schizophrenia (SCZ) or psychopathy, which portends a poorer overall prognosis^5^. Despite advances in our clinical knowledge of ASPD, comparatively little is known about its neurobiology.

A key feature of ASPD is high endorsement of reactive or impulsive aggression, which is aggression enacted in response to provocative or threatening stimuli. The neural circuit that mediates reactive aggression has been localized to the amygdala, hypothalamus, and periaqueductal gray^6^. A combination of increased, hyperactive limbic (e.g., amygdala) structures in concert with decreased orbitofrontal cortex (OFC) activity is a highly-regarded hypothesis of the mechanism underlying reactive aggression^7^. Studies of reactively aggressive populations, including individuals with intermittent explosive disorder^8^, impulsively aggressive spouse abusers^9^, and reactively aggressive violent offenders^10^ all report increased amygdala responsiveness to threatening stimuli. Since the OFC and amygdala share numerous reciprocal connections that influence impulsivity^11^, there is a need to prioritize research of these regions in neurobiological studies of ASPD.

Our understanding of the molecular underpinnings of ASPD is particularly limited. To our knowledge, there are no postmortem studies of individuals with the clinical diagnosis. Moreover, extant brain positron emission tomography (PET) studies have primarily focused on monoaminergic neurotransmission in relation to the diagnosis or characteristics of the disorder^12^. Fortunately, new positron emission tomography (PET) radiotracers have been developed that can radiolabel core constituents of the endocannabinoid system (ECS) in vivo^13^. This advance is noteworthy, since the ECS has been implicated in the pathophysiology of ASPD and aggressive behavior^14^, although data linking the ECS to impulsive aggression have been mixed and largely limited to animal models^15^.

Composed of cannabinoid receptors, endocannabinoid (EC) ligands, and enzymes that regulate EC function, a chief function of the ECS is to modulate cognitive processes and social behaviors^16^. One molecule of interest is the enzyme fatty acid amide hydrolase (FAAH). Located post-synaptic to the cannabinoid 1 receptor (CB1), FAAH metabolizes the endocannabinoid N-arachidonoylethanolamine (anandamide; AEA). AEA is an EC that principally binds to CB1^17^. In a unique signaling pathway, AEA is synthesized on-demand in post-synaptic neurons before engaging in retrograde feedback onto pre-synaptic CB1^18^. Molecules that regulate AEA are thus able to indirectly control CB1 signaling and presumably modulate socioemotional behaviors, including aggression^19^.

Boileau and colleagues were the first to discover that a single nucleotide polymorphism of the *FAAH* gene (rs324420), which involves transversion of a cytosine residue to the nucleoside adenine (C385A), affects the kinetics of [^11^C]CURB, a PET radiotracer that can quantify FAAH density. Both C/A and A/A genotypes have lower production of in vivo brain FAAH expression relative to those with the C/C genotype^20^. Interestingly, in a sample of Spanish males with alcohol dependence, an association between ASPD and greater frequency of the C385A genotype emerged, suggesting lower brain levels of FAAH in ASPD^14^. Lower brain FAAH expression is consistent with the notion of higher brain levels of AEA and subsequently greater CB1 activation. Here, we suggest that hyperactivity of the amygdala observed in reactively aggressive populations may result from increased CB1 neurotransmission as a result of lower FAAH density. On the contrary, increased OFC activity may stem in part from higher FAAH levels and decreased CB1 activation, consistent with recent findings of another study that found elevated PFC FAAH density in an aggressive population^21^. Application of [^11^C]CURB PET, a radioligand with high specificity for the FAAH enzyme^13^, could extend functional magnetic resonance imaging findings in reactively aggressive ASPD to contribute plausible neurochemical mechanisms explaining the disorder and pathological core traits.

Our primary objective was to assay FAAH expression in the amygdala and OFC of reactively aggressive violent offenders with ASPD, although we also conducted a whole-brain analysis. We selected for primary analysis the amygdala and OFC, given their putative involvement in the commission of reactive aggression and the fact that they have shown structural and functional abnormalities in ASPD and antisocial populations^22^. We included ASPD individuals with and without comorbid SCZ to increase the generalizability of results. For example, although ASPD is very common in forensic psychiatric patients who have primary psychotic disorders, the comorbid conditions have rarely, if ever, been investigated in PET investigations. We hypothesized that amygdala FAAH expression would be lower in ASPD with reactive aggression and higher in the OFC of reactively aggressive ASPD. We further hypothesized that measures of impulsivity and aggression would correlate with FAAH density in the brain regions of interest.

## Patients and methods

All participants provided written informed consent after all study components were fully explained to them. The Research Ethics Board of the Centre for Addiction and Mental Health (CAMH) in Toronto, Ontario, Canada, approved all procedures of this investigation.

### Participants

Sixteen patients with ASPD (ASPD-SCZ = 11; ASPD + SCZ = 5) and a history of violent offending and 16 control participants (healthy controls = 11; SCZ = 5) with no history of violent offending completed the investigation. The sample size chosen to detect a pre-specified effect was based on similar sample sizes of recent PET experiments^23^. Inclusion and exclusion criteria were pre-determined. Experimental participants and controls were sex-matched (all male). All diagnoses were verified according to results from the Structured Clinical Interview for DSM-IV Axis II Disorders (SCID-II)^24^ and Structured Clinical Interview for DSM-IV Axis I Disorders (SCID-I)^25^ (for the ASPD and healthy controls) by trained raters. In addition, ASPD diagnoses were reviewed and confirmed by a forensic psychiatrist experienced in the assessment and treatment of personality disorders (NJK).

### ASPD-SCZ

ASPD-SCZ participants were recruited from the local Toronto community and correctional centers. Exclusion criteria for the ASPD-SCZ participants included a current major depressive episode (MDE); history of mania, hypomania, or psychotic illness; and diagnosis of substance abuse or dependence in the past 12 months as confirmed by the SCID-I. The use of psychotropic medications or herbs in the past three months was also exclusionary. One ASPD-SCZ participant was a smoker. For all study groups (ASPD-SCZ; ASPD + SCZ; SCZ; and healthy controls), neurological illness; head trauma; positive drug screen for drugs of abuse, including cannabis, on scan and assessment days; and contraindications to safe magnetic resonance imaging (MRI) scanning also precluded participation.

### ASPD + SCZ

ASPD + SCZ participants were recruited from forensic psychiatry inpatient and outpatient services at CAMH. Exclusion criteria for the ASPD + SCZ participants included an MDE and/or substance use disorder in the past 12 months. Three ASPD + SCZ participants were current smokers. For all ASPD participants (ASPD-SCZ and ASPD + SCZ), the urine drug screen utilized was Rapid Response^TM^, Drugs of Abuse Test Panel (BTNX Inc., Markham, Ontario, Canada). It tests for the presence of opiates, phencyclidine, barbiturates, benzodiazepines, tricyclic antidepressants, amphetamines, tetrahydrocannabinol, and methadone.

### SCZ

SCZ participants were recruited from outpatient programs at a tertiary care psychiatric center. They were selected from a larger group of SCZ patients, because they had not used illicit substances, including cannabis. Exclusion criteria for the SCZ participants included a positive drug screen at assessment and scanning days and a diagnosis of substance abuse or dependence. None of the SCZ participants were smokers. The drug screen used for SCZ participants was a urine immunoassay administered at the CAMH laboratory that tested for the presence of ethanol, methadone, benzodiazepines, tetrahydrocannabinol, opiates, and cocaine metabolites.

### Healthy controls

Healthy controls had no history of psychiatric illness according to SCID-I and SCID-II reports. They were recruited from the community. All were non-smokers. The drug screen used for healthy participants was manufactured by BTNX (BTNX Inc., Markham, Ontario, Canada). It tests for the presence of cocaine, amphetamines, opiates, oxycodone, methadone, 3,4-methylenedioxymethamphetamine, benzodiazepines, ketamine, tetrahydrocannabinol, and tricyclic antidepressants.

All study participants were asked to refrain from using alcohol the night before and the day of PET scanning.

### Image acquisition and analysis

Each participant completed one [^11^C]CURB PET scan at the CAMH Brain Health Imaging Centre. Radiosynthesis of [^11^C]CURB has been previously described^26^. Participants underwent PET utilizing a three-dimensional HRRT brain tomograph (CPS/Siemens, Knoxville, TN, USA). For the duration of the scan, participants lay on their backs and wore a thermoplastic mask to reduce movement. A transmission scan was conducted followed by injection of 370 ± 40 MBq (10 ± 1 mCi) of [^11^C]CURB^27^. An advantage of [^11^C]CURB is its high specific binding^13^. Brain radioactivity was computed during sequential frames of increasing duration, and the scan time was 60 min. PET images were next re-constructed using a filtered back-projection algorithm with a HANN filter at Nyquist cutoff frequency^28^. Arterial samples were sampled continuously for the first 22.5 min with an automatic blood sampling system (Model PBS-101, Veenstra Instruments, Joure, The Netherlands) after [^11^C]CURB injection. Radioactivity in whole blood and plasma (1500 relative centrifugal force, 5 min) was counted utilizing a Packard Cobra II or Wizard 2480 γ-counter (Packard Instrument Co., Meridian, CT, USA) cross-calibrated with the PET system. The concentration of parent radioligand and its metabolites was measured in each manual sample (except for the one at 15 min) as previously validated^26^. Blood-to-plasma radioactivity ratios were fitted utilizing a biexponential function and parent plasma fraction using a Hill function. A dispersion- and metabolite-corrected arterial plasma input function was generated as previously described^26^.

Each participant received a standard proton-density weighted brain MRI scan (TE = 17, TR = 6000, FOV = 22 cm, matrix = 256 × 256, slice thickness = 2 mm; number of excitations = 2) acquired on a Discovery MR750 3 T MRI scanner (General Electric, Milwaukee, WI, USA) for region of interest (ROI) delineation. ROIs were generated automatically using in-house software (ROMI) as previously reported^29^. Time-activity curves were acquired over 60 min. in each of the ROIs and analyzed by a two-tissue compartment model with irreversible binding to the second component^26^. FAAH binding was quantified using the composite parameter λ*k*_3_ (λ = *K*_1_/*k*_2_).

### *FAAH* polymorphism genotyping

For all study participants, the *FAAH* rs324420 variant was genotyped according to the manufacturer’s directions for a TaqMan SNP Genotyping assay (ID C_1897306_10; Life Technologies, Burlington, Canada) on a ViiA7 instrument (Life Technologies, Burlington, Canada) using 20 ng total genomic DNA template, Perfecta FastMix II (Quantabio, Beverly, MA, USA), in a total reaction volume of 10 μL as previously performed^20^.

### Instruments

ASPD subjects completed the Urgency, Premeditation, Perseverance, Sensation Seeking, Positive Urgency Impulsive Behavior Scale (UPPS-P)^30^ to quantify facets of impulsivity; the Buss-Durkee Hostility Index (BDHI)^31^ to assess levels of hostility and aggression; and the Reactive-Proactive Aggression Questionnaire^32^ to measure levels of reactive aggression; the Psychopathy Checklist: Screening Version (PCL:SV)^33^ and Triarchic Psychopathy Measure (TriPM)^34^ to gauge the presence of psychopathic traits; and the State-Trait Anxiety Inventory (STAI)^35^ to measure anxiety symptoms.

### Statistical analysis

Sample demographic and clinical information were compared between groups using chi-square tests, independent samples *t*-tests, and independent samples Mann–Whitney *U* tests. All tests of significance were two-sided. The Shapiro–Wilks test was used to assess the normality of data for all comparisons of continuous data. Non-parametric tests were utilized where data violated the assumptions of normality. To compare [^11^C]CURB λ*k*_3_ between groups (ASPD + SCZ and ASPD-SCZ versus SCZ and healthy controls), we employed multivariate analysis of covariance (MANCOVA) with ASPD diagnosis as a between-subjects factor and genotype as a covariate. The main model that was used to test our primary hypothesis included two ROIs: OFC and amygdala. Main effects were analyzed by univariate ANOVA with a *p*-value < 0.05 indicating significance. Our secondary model tested the whole brain, including anterior cingulate cortex (ACC), temporal cortex, insula, hippocampus, thalamus, ventral striatum, dorsal putamen, dorsal caudate, and cerebellum (11 ROIs). Bonferroni corrections were applied (0.05/9 secondary regions = 0.0056) to correct for multiple comparisons.

Partial correlations between UPPS-P and BDHI scores with FAAH binding in our ROIs controlling for FAAH genotype were planned a priori and were not adjusted for multiple comparisons. For partial correlations examining psychopathic traits and anxiety symptoms, Bonferroni correction was applied. Data in Table 1 are presented as means and standard deviations.

**Table 1:** Clinical and demographic variables.

	ASPD^π^ (*n* = 16)	Controls (*n* = 16)	Statistics	*p*-value
Age (years)^a^	35.6 ± 9.4	30.0 ± 9.5	*t* = 1.7	0.10
Sex (M/F)	16/0	16/0	/	/
Ethnicity^b^	/	/	*χ*^2^ = 9.5	0.090
Caucasian (#)	10	7	/	/
Black (#)	3	3	/	/
Hispanic (#)	2	0	/	/
Aboriginal (#)	1	0	/	/
Asian (#)	0	6	/	/
Education (years)^a^	13.0 ± 2.3	16.0 ± 2.2	*t* = −3.5	0.002
Taking antipsychotics (schizophrenia subjects)	5	5	/	/
Genotype (CC or CA/AA)^b^	9/7	13/3	*χ*^2^ = 2.3	0.13
Body mass index^a,c^	26.7 ± 3.5	25.3 ± 4.3	*t* = 1.0	0.33
BDHI^§^				
Assault	6.4 ± 2.3	/	/	/
Indirect	6.4 ± 2.0	/	/	/
Irritability	8.1 ± 2.7	/	/	/
Negativity	3.5 ± 1.4	/	/	/
Resentment	5.6 ± 2.4	/	/	/
Suspicion	5.9 ± 2.4	/	/	/
Verbal	10.3 ± 2.2	/	/	/
Guilt	5.3 ± 2.2	/	/	/
Total	46.1 ± 12.0	/	/	/
RPAQ^Δ^				
Reactive aggression	13.8 ± 4.5	/	/	/
UPPS-P (mean of total responses)^ǂ^				
Negative urgency	2.8 ± 0.4	/	/	/
Lack of premeditation	2.3 ± 0.7	/	/	/
Lack of perseverance	2.2 ± 0.6	/	/	/
Sensation seeking	2.5 ± 0.7	/	/	/
Positive urgency	2.5 ± 0.7	/	/	/
Total	15.2 ± 2.5	/	/	/
PCL:SV^Φ^				
Interpersonal/affective	8.1 ± 5.0	/	/	/
Antisocial/deviance	8.5 ± 2.1	/	/	/
Total	13.0 ± 3.8	/	/	/
TriPM^Ω^				
Boldness	31.9 ± 9.2	/	/	/
Meanness	22.7 ± 12.8	/	/	/
Disinhibition	38.4 ± 10.3	/	/	/
Total	93.1 ± 24.4	/	/	/
STAI^θ^				
Trait	48.0 ± 12.2	/	/	/
State	42.7 ± 14.8	/	/	/
Tracer specific activity (mCi/μmol)^d^	3683.2 ± 2035.9	3213.9 ± 1476.8	/	0.59
Mass injected (μg)^d^	2.0 ± 1.5	1.7 ± 0.8	/	0.72

^a^Independent samples *t*-test.

^b^Chi-square test.

^c^1 ASPD participant’s BMI data is missing.

^d^Independent samples Mann–Whitney U test.

^π^Antisocial personality disorder.

^§^Buss-Durkee Hostility Inventory.

^Δ^Reactive-Proactive Aggression Questionnaire.

^ǂ^Urgency, Premeditation, Perseverance, Sensation Seeking, Positive Urgency Impulsive Behavior Scale.

^Φ^Psychopathy Checklist: Screening Version.

^Ω^Triarchic Psychopathy Measure.

^θ^State-Trait Anxiety Inventory.

## Results

### Subject characteristics

Participants’ clinical and demographic information is reported in Table 1.

Information on comorbid psychiatric conditions is presented in Supplementary Table 1, and data on ASPD subjects’ violent offenses are presented in Supplementary Table 2.

Participants were aged 18–49 years. None of the participants screened positive for drugs of abuse, including cannabis. Healthy participants (e.g., no ASPD, no SCZ) and community participants with SCZ had previously participated in other PET experiments^36,37^. Groups did not differ in terms of age, ethnicity, body mass index, or number of participants with the C385A *FAAH* genotype. The ASPD group had fewer years of education compared with the control group. Therefore, education level was included as a covariate in the multivariate analyses, as the necessity of matching or controlling for this variable, potentially as a proxy for IQ, has been previously identified in studies of antisocial populations^38^.

### Comparison of [^11^C]CURB λ*k*_3_ in ASPD and control groups

We observed an overall effect of ASPD diagnosis on [^11^C]CURB λ*k*_3_ across the two hypothesized regions of interest (F_2,26_ = 4.5, *p* = 0.020). Using the effect of ASPD diagnosis in the MANCOVA for our two hypothesized ROIs (OFC and amygdala), results revealed that ASPD subjects had 12.5% lower amygdala [^11^C]CURB λ*k*_3_ compared with the control subjects (0.12 ± 0.033 versus 0.15 ± 0.029, F_1,27_ = 7.2, *p* = 0.013). When we re-ran the analysis without incorporating education as a covariate, results remained significant (*p* = 0.046). However, there was no significant difference in OFC [^11^C]CURB λ*k*_3_ between groups (11.7% reduction; 0.12 ± 0.028 versus 0.14 ± 0.021, F_1,27_ = 1.7, *p* = 0.21). There were no significant interactions. Genotype remained significant.

**Fig. 1:**
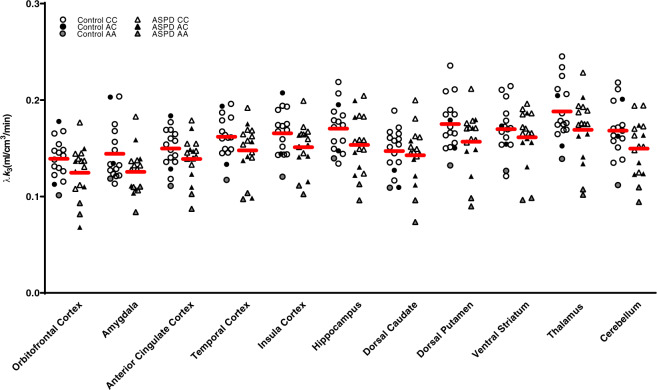
Lower amygdala fatty acid amide hydrolase λ*k*_3_ in antisocial personality disorder. After controlling for genotype, antisocial personality disorder participants were found to have lower fatty acid amide hydrolase density in the amygdala compared with controls (*p* = 0.013).

No significant effect of group was ascertained when we sampled the 11 ROIs for the whole-brain analysis (F_11,17_ = 1.4, *p* = 0.27; percentage of decreased FAAH binding in ASPD for each ROI: 10.6–12.5%). None of the regions survived Bonferroni correction. These results are presented graphically in Fig. 1. Statistics for secondary ROIs are presented in Supplementary Table 3. As expected, there was a main effect of genotype for the analysis (*p* = 0.003). There were no significant interactions.

### Partial correlations between [^11^C]CURB λ*k*_3_ and hostility, aggression, impulsivity, psychopathic traits, and anxiety

**Fig. 2:**
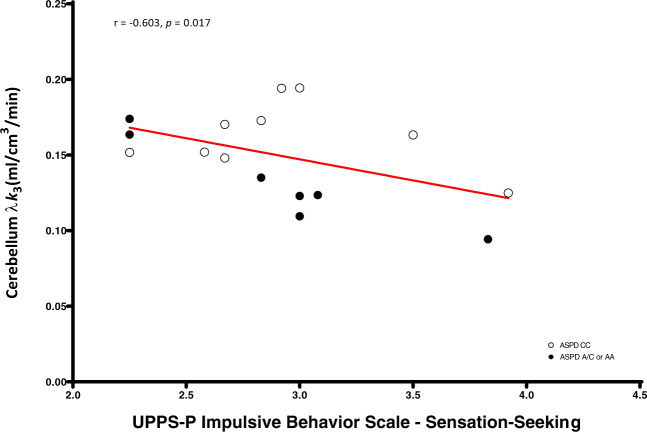
Cerebellum fatty acid amide hydrolase λ*k*_3_ is negatively correlated with UPPS-P Impulsive Behavior Scale—sensation-seeking in antisocial personality disorder. After controlling for genotype, antisocial personality disorder participants with lower cerebellar fatty acid amide hydrolase density endorsed greater sensation-seeking impulsivity.

**Fig. 3:**
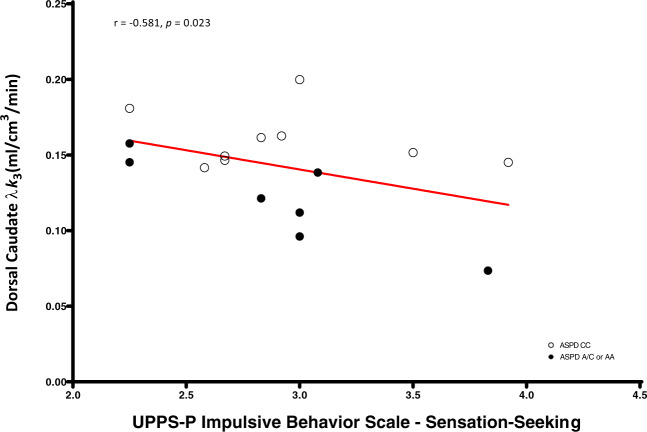
Dorsal caudate fatty acid amide hydrolase λ*k*_3_ is negatively correlated with UPPS-P Impulsive Behavior Scale—sensation-seeking in antisocial personality disorder. After controlling for genotype, antisocial personality disorder participants with lower dorsal caudate fatty acid amide hydrolase density endorsed greater sensation-seeking impulsivity.

**Fig. 4:**
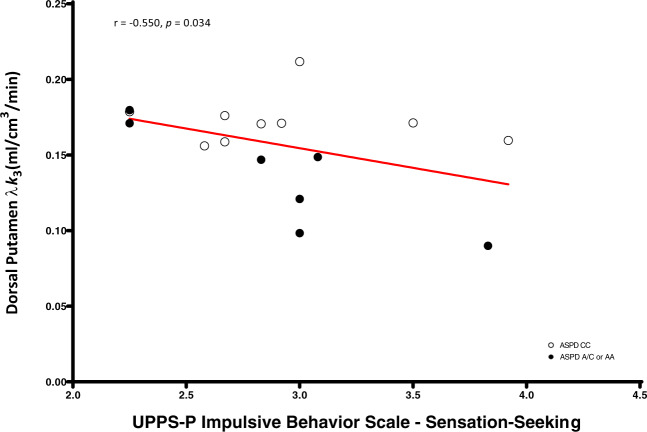
Dorsal putamen fatty acid amide hydrolase λ*k*_3_ is negatively correlated with UPPS-P Impulsive Behavior Scale–sensation-seeking in antisocial personality disorder. After controlling for genotype, antisocial personality disorder participants with lower dorsal putamen fatty acid amide hydrolase endorsed greater sensation-seeking impulsivity.

**Fig. 5:**
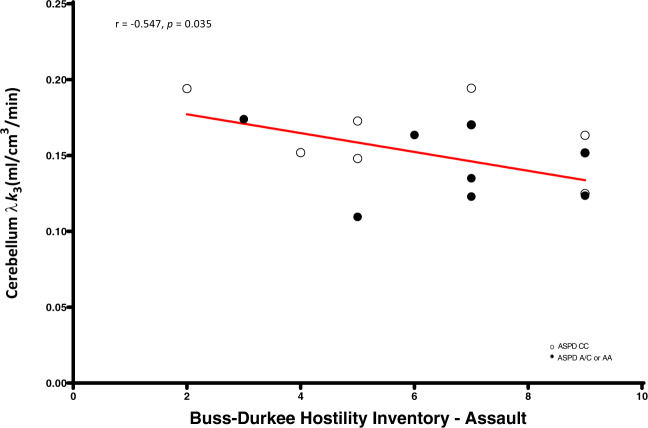
Cerebellum fatty acid amide hydrolase λ*k*_3_ is negatively correlated with Buss-Durkee Hostility Inventory—assault in antisocial personality disorder. After controlling for genotype, antisocial personality disorder participants with lower cerebellar fatty acid amide hydrolase density endorsed greater assaultive behavior.

We found significant negative correlations between dorsal caudate (*r* = −0.58, *p* = 0.023), dorsal putamen (*r* = −0.55, *p* = 0.034), and cerebellum (*r* = −0.60, *p* = 0.017) [^11^C]CURB λ*k*_3_ with sensation-seeking impulsivity, controlling for genotype, and a significant negative association between cerebellum [^11^C]CURB λ*k*_3_ (*r* = −0.54, *p* = 0.035) and assaultive behavior, controlling for genotype. None of the partial correlations between the 11 ROI [^11^C]CURB λ*k*_3_ values and reactive aggression, psychopathic traits, or anxiety were significant (all *p*-values > 0.05) (Figs. 2–5).

## Conclusion

Consistent with our main hypothesis, we found that amygdala [^11^C]CURB λ*k*_3_ was lower in ASPD versus participants without ASPD. We also discovered that sensation-seeking impulsivity in the ASPD group was negatively correlated with dorsal caudate, dorsal putamen, and cerebellum [^11^C]CURB λ*k*_3_, while cerebellum [^11^C]CURB λ*k*_3_ was negatively associated with assaultive behavior. To our knowledge, this PET study is the first to sample a target beyond the monoaminergic systems in ASPD. Although our sample is relatively small and the robustness of the statistical tests is modest, we believe that these findings can potentially provide a novel neurochemical mechanism to partially explain core deficits in ASPD related to reactive aggression and impulsivity.

We observed decreased amygdala [^11^C]CURB λ*k*_3_. However, none of the other regions sampled had significantly lower [^11^C]CURB λ*k*_3_ values. Lower amygdala FAAH expression is consistent with decreased FAAH expression observed across the brain in other externalizing conditions, such as alcohol and substance use disorders^37,39^. Our study was likely underpowered to detect differences in all ROIs. We suggest that alterations in [^11^C]CURB λ*k*_3_ may be disorder-specific depending on whether internalizing or externalizing spectra are more prominent. For example, we would expect that primarily internalizing disorders, including major depressive disorder or posttraumatic stress disorder, would display higher brain FAAH density. Conducting [^11^C]CURB PET experiments of FAAH density in these and other psychiatric disorders could help answer this question.

Mounting evidence points to the role of the amygdala in stress-related psychiatric disorders and how the amygdala exhibits plasticity in response to acute stressors. This line of inquiry has implications for understanding the mechanisms of reactive aggression in ASPD. Interestingly, administration of FAAH inhibitors to animal models prevents morphological changes of the amygdala typically observed following stressful insults^40^, which might help explain the impaired stress response and propensity to respond with reactive aggression in ASPD. As a result, we suggest that lower levels of amygdala FAAH may impair how ASPD individuals process negative emotional stimuli, such as fearful or angry facial expressions. Mechanistically, lower FAAH levels should lead to greater CB1 neurotransmission, which may be responsible for hyper-reactivity of the amygdala in response to stressful, threatening situations. We speculate that lower levels of amygdala FAAH expression may prevent persons with ASPD from responding appropriately to stress, making them more inclined to engage in reactive aggressive and impulsive behavior.

A second main finding is that assaultive aggression was negatively correlated with cerebellum [^11^C]CURB λ*k*_3_. We do note, however, that the cerebellum was not an a priori hypothesized ROI. Alterations in cerebellum structure and function have been previously reported in violent offenders^41–44^. Moreover, converging lines of neuroimaging evidence link the cerebellum to moral behavior and aggression^45^. Interestingly, the cerebellum has very high CB1 density^46,47^, and in the cerebellum, FAAH-positive cell bodies abut axon terminals containing CB1 receptors^48^. Thus, the ECS is highly expressed in cerebellum and contributes to cerebellar function^49,50^. There is elevated aggressive behavior in CB1 knockout mice^51^, and cannabinoid agonists are being investigated in clinical trials for treating agitation and aggression in Alzheimer’s patients^52^. Still, little is known about the role of cerebellar ECS in aggressive behavior. Hence, there is a need for more investigation to better understand the relationship between cerebellar FAAH and aggressive behavior.

The negative correlations reported between striatal/cerebellar FAAH binding and impulsivity mirror previous results of lower FAAH binding and higher trait impulsivity among individuals with cannabis use disorder during early abstinence^39^. There is high heritability of impulsivity in externalizing conditions, such as substance use disorders and ASPD^53^. Thus, genes regulating FAAH availability may be particularly important for impulsive phenotypes in externalizing conditions. Sensation-seeking impulsivity, in particular, is elevated among individuals who perpetrate antisocial behavior^54^. The neural substrate of impulsivity includes dysfunctional fronto-cerebellar connections^55^ and faulty fronto-striatal networks, consistent with the regions identified in the current study^56^. Since animal work suggests that FAAH deficiency increases motivations for rewards and sensation seeking^57^, low FAAH expression in these regions may predispose to impulsivity among individuals with high genetic predisposition for this trait.

There are currently no FDA-approved psychotherapeutics for the treatment of ASPD. Moreover, typically applied agents, such as antidepressants, mood stabilizers, anticonvulsants, and antipsychotics, have not been subject to rigorous testing. Since our preliminary data tentatively indicate that low FAAH may be implicated in the pathophysiology of ASPD, impulsivity, and aggression, interventions capable of increasing FAAH activity in a regionally-specific manner could possibly emerge as viable treatment options for ASPD. Consistent with this notion, leptin is a peripheral, adipose-derived hormone that functions as an indicator to the central nervous system (CNS) of energy storage^58^. There are several isoforms of the leptin receptor, which curb caloric intake, and which are widely distributed in the CNS. For example, the long form of the leptin receptor is expressed in Purkinje cells and dentate nucleus of the cerebellum as well as the amygdala among other regions in the human brain^59^. Leptin has recently been shown to increase brain FAAH levels in a knock-in mouse model of low FAAH activity (C385A polymorphism, rs324420)^60^. Similar to claims that reinstating FAAH activity to suppress AEA activity could underlie new anti-obesity treatments^60^, these same postulated therapeutics may also be effective for reducing reactive aggression and impulsivity. Testing such therapeutics on individuals with ASPD could open up new avenues for treatment.

We recently reported that there were no significant group differences between amygdala FAAH expression in borderline personality disorder (BPD) and healthy controls^21^. Given some similarities in clinical characteristics between BPD and ASPD, the contrasting effects could suggest at least two interpretations. One possibility is that one of the findings is spurious. A second possibility is that we have identified a brain feature that may account for differences between BPD and ASPD. We favor the latter interpretation, as molecular differences have previously been reported in the two conditions. For example, in one PET study, brain density of monoamine oxidase-A (MAO-A), an enzyme that degrades catecholamine neurotransmitters in the CNS, was greater in the PFC of BPD compared with healthy controls^61^. MAO-A total distribution volume also correlated with depression and suicidality in the BPD participants. Conversely, in another PET study of ASPD that also assayed brain MAO-A total distribution volume, orbitofrontal MAO-A levels were lower in ASPD compared with a control group composed of healthy individuals and some with alcohol dependence^62^. Therefore, we tentatively suggest that differential amygdala FAAH expression in ASPD and BPD could reflect a biomarker that is diagnosis-specific.

We note several limitations of the present investigation. First, the study design cannot delineate whether lower [^11^C]CURB λ*k*_3_ in ASPD is a state or trait marker of psychopathology. Longitudinal designs that measured amygdala [^11^C]CURB λ*k*_3_ at several time points could determine whether this biomarker is malleable to clinical improvement. Second, we acknowledge that our sample size is relatively small. Given some evidence that antisocial populations with and without comorbid anxiety disorders may manifest different brain correlates^63^, future PET studies with larger samples may consider tackling the question of whether amygdala [^11^C]CURB λ*k*_3_ differs according to the presence or absence of anxiety disorders in violent offenders. Third, we did not measure serum levels of endocannabinoids such as AEA or the *N*-acylethanolamides *N*-palmitoylethanolamine and *N*-oleoylethanolamine. While measuring plasma concentrations of FAAH substrates could potentially identify peripheral biomarkers of brain FAAH activity, the relationship between brain and peripheral concentration of ECs is currently obscured due to excess physiological noise^64^. Fourth, we were unable to match participants from each group on the exact *FAAH* polymorphism (rs324420), although we found no difference in the proportion between groups and we also controlled for genotype. While the frequency distribution of the C385A genotype in ASPD is unknown in the general population, preliminary evidence suggests that it could be higher in individuals with ASPD^14^. Fifth, although we found group differences in the a priori ROI (amygdala), the correlations were detected in other brain regions. We draw attention to this fact, as these findings would not meet statistical significance under most corrections. Thus, these results should be viewed with caution. Sixth, although there is a modest positive correlation between intelligence test scores and academic achievement (data which we had available)^65,66^, we did not have IQ scores for all participants. We also found group differences in educational achievement; it is conceivable that group differences may also have been observed if IQ data had been available for all subjects. Future studies should endeavor to include IQ as a measure in PET studies of antisocial offenders, given that components of the endocannabinoid system may be linked to IQ, as has been shown for serotonin levels and cognition^67,68^. Finally, our sample of ASPD participants were all convicted of their violent crimes and, therefore, may differ in important ways from other ASPD individuals who offend but successfully evade the law.

In conclusion, ASPD is a very common yet understudied condition whose pathological features may be amenable to novel psychotherapeutics. PET investigations of ASPD with novel radioligands are, therefore, critical to advance the field. We found that amygdala FAAH brain activity was lower in ASPD, a finding that resonates with previous studies of externalizing populations. Higher impulsivity and greater aggression were additionally associated with lower FAAH [^11^C]CURB λ*k*_3_ in a region-specific manner. However, these results must be viewed with caution, given the small sample size and modest results.

## Supplementary information

Supplemental Table 1

Supplemental Table 2

Supplemental Table 3
