# Supplementary material for: Lower amygdala fatty acid amide hydrolase in violent offenders with antisocial personality disorder: an [11C]CURB positron emission tomography study
Source: Transl Psychiatry. 2021 Jan 18;11:57. doi: 10.1038/s41398-020-01144-2 (PMC7814116; doi:10.1038/s41398-020-01144-2)
Supplement: Supplementary file 1 — Supplemental Table 1 [file 41398_2020_1144_MOESM1_ESM.docx]

**Supplementary Table 1**

**Comorbid Psychiatric Diagnoses**  ASPD Controls

*n* = 16 *n* = 16

____________________________________________________________________________________________________

Lifetime major depressive disorder (%) 10 18.9% (all SCZ subjects)

Lifetime dysthymic disorder (%) 0 /

Current panic disorder (%) 6.3 6.3% (SCZ subject)

Current agoraphobia (%) 0 /

Current specific phobia (%) 6.3 /

Current social phobia (%) 0 /

Current generalized anxiety disorder (%) 12.5 6.3% (SCZ subject)

Current obsessive compulsive disorder (%) 12.5 /

Current posttraumatic stress disorder (%) 18.8 /

Previous alcohol use disorder (%) 37.5 /

Previous cannabis use disorder (%) 25 /

Previous opioid use disorder (%) 6.3 /

Previous sedative/hypnotic use disorder (%) 6.3 /

Previous stimulant use disorder (%) 6.3 /

Previous cocaine use disorder (%) 12.5 /

Previous hallucinogen use disorder (%) 6.3 /

Previous polysubstance dependence (%) 6.3 /

Current somatization disorder (%) 0 /

Current pain disorder (%) 0 /

Current undifferentiated somatoform disorder (%) 0 /

Current hypochondriasis (%) 0 /

Current body dysmorphic disorder (%) 0 /

Current anorexia nervosa (%) 0 /

Current bulimia nervosa (%) 0 /

Current eating disorder not otherwise specified (%) 0 /

Current paranoid personality disorder (%) 0 /

Current schizoid personality disorder (%) 12.5 /

Current schizotypal personality disorder (%) 0 /

Current borderline personality disorder (%) 6.3 /

Current histrionic personality disorder (%) 0 /

Current narcissistic personality disorder (%) 25 /

Current avoidant personality disorder (%) 6.3 /

Current obsessive compulsive personality disorder (%) 6.3 /

Current dependent personality disorder (%) 12.5 /

Schizophrenia (%) 31.3 31.3
