# Supplementary material for: Lower amygdala fatty acid amide hydrolase in violent offenders with antisocial personality disorder: an [11C]CURB positron emission tomography study
Source: Transl Psychiatry. 2021 Jan 18;11:57. doi: 10.1038/s41398-020-01144-2 (PMC7814116; doi:10.1038/s41398-020-01144-2)
Supplement: Supplementary file 2 — Supplemental Table 2 [file 41398_2020_1144_MOESM2_ESM.docx]

**Supplementary Table 2**

**Violent Offenses in ASPD Participants**

________________________________________________________________________________________

ASPD 1 Assault with weapon

ASPD 2 Assaults, Robberies

ASPD 3 Assaults, Uttering death threats

ASPD 4 Assaults

ASPD 5 Assaults

ASPD 6 Assaults

ASPD 7 Assault with weapon, Robbery, Assault with forcible confinement

ASPD 8 Assaults

ASPD 9 Assault, Assault with weapon

ASPD 10 Sexual assault, Assault

ASPD 11 Attempt murder, Aggravated assault, Assaults

ASPD 12 Aggravated assault, Assaults, Robbery, Uttering threats

ASPD 13 Aggravated sexual assault, Assaults, Robbery

ASPD 14 Assaults, Uttering death threats

ASPD 15 Aggravated assault, Assault with weapon, Robbery

ASPD 16 Aggravated assault, Assaults

__________________________________________________________________________________________
