# Supplementary material for: Lower amygdala fatty acid amide hydrolase in violent offenders with antisocial personality disorder: an [11C]CURB positron emission tomography study
Source: Transl Psychiatry. 2021 Jan 18;11:57. doi: 10.1038/s41398-020-01144-2 (PMC7814116; doi:10.1038/s41398-020-01144-2)
Supplement: Supplementary file 3 — Supplemental Table 3 [file 41398_2020_1144_MOESM3_ESM.docx]

**Supplementary Table 3**

**Statistics for Secondary Regions of Interest**

Secondary Regions of Interest (ASPD vs. Controls) *F* Degrees of Freedom *p-*value

Anterior Cingulate Cortex 1.0 1, 28 0.33

Insula Cortex 2.1 1, 28 0.16

Temporal Cortex 1.4 1, 28 0.25

Hippocampus 1.6 1, 28 0.21

Dorsal Caudate 0.40 1, 28 0.53

Dorsal Putamen 1.4 1, 28 0.25

Ventral striatum 0.13 1, 28 0.72

Thalamus 1.3 1, 28 0.26

Cerebellum 2.3 1, 28 0.14
